# Editorial: New frontiers in heart failure therapy: mechanisms, efficacy, and challenges

**DOI:** 10.3389/fcvm.2026.1902385

**Published:** 2026-07-07

**Authors:** Tzu-Hurng Cheng, Youhua Wang, Dobrin Vassilev

**Affiliations:** 1 Department of Biochemistry, School of Medicine, China Medical University, Taichung, Taiwan; 2 Cardiovascular Research & Cardiovascular Department, Longhua Hospital, Shanghai University of Traditional Chinese Medicine (TCM), Shanghai, China; 3 Faculty of Public Health and Health Care, Ruse University, Ruse, Bulgaria

**Keywords:** drug therapy, heart failure, molecular mechanisms of pharmacological action, translational medical research, treatment outcome

Heart failure remains a major global health burden, characterized by complex pathophysiology and persistent gaps in therapeutic outcomes. Despite advances in pharmacological and device-based interventions, patients continue to face high rates of hospitalization, impaired quality of life, and significant mortality. The Research Topic New Frontiers in Heart Failure Therapy: Mechanisms, Efficacy, and Challenges was established to address these unmet needs by integrating perspectives from molecular biology, translational research, and clinical practice. The 18 contributions, authored by 122 researchers, collectively provide new insights into disease mechanisms, evaluate therapeutic efficacy, and highlight ongoing challenges in management. Mechanistic investigations have advanced understanding of disease progression. Liu et al. demonstrated that early treatment with nootkatone prevents pressure overload–induced ventricular remodeling and heart failure. Du et al. showed that Poge heart-saving decoction ameliorates heart failure by suppressing apoptosis and fibrosis via regulation of the PI3 K/AKT pathway. Yongjie et al. synthesized evidence on hypoxia-driven mitochondrial and ion channel dynamics, introducing hypoxia-inducible factor (HIF)-mediated hierarchical adaptation as a novel paradigm in heart failure pathogenesis. Complementary work addressed oxidative stress Zhang et al., inflammasome activation in heart failure with preserved ejection fraction (HFpEF) Yu et al., and genetic determinants of disease progression Wang et al. Collectively, these contributions expand the mechanistic landscape, identifying molecular targets with therapeutic potential. Therapeutic evaluations highlight both established and emerging strategies. Studies reassessed interventions such as cardiac resynchronization therapy Małek-Elikowska et al. and beta-blockers Xie et al. Comparative analyses of angiotensin receptor–neprilysin inhibition provided evidence for optimizing pharmacological regimens. Novel agents, including vericiguat Yongjie et al. and finerenone Zhao et al. and Peng et al., were highlighted for their promise in patients with reduced or preserved ejection fraction. Reviews emphasized advances in mineralocorticoid receptor antagonists in the era of sodium-glucose cotransporter-2 (SGLT2) inhibitors and glucagon-like peptide-1 (GLP-1) receptor agonists. Opinion contributions stressed the value of combining pharmacological and device-based approaches Li et al., pointing toward integrated strategies tailored to patient needs. Clinical perspectives underscore the realities of care delivery. Investigations addressed drug resistance Zhao et al., adverse effects Wang et al., and inequities in treatment access. Several authors stressed the importance of tailoring therapy to individual patient characteristics Kumar et al., while perspectives on precision medicine and interdisciplinary collaboration reinforced the need to align basic science, clinical practice, and health policy. Additional contributions explored innovative therapeutic avenues, including bioactive peptide-peptoid hybrids Kumar et al., long-acting relaxin analogues Wołowiec et al., and traditional decoctions such as Ershen Zhenwu. Altogether, this collection offers a comprehensive synthesis of mechanistic, therapeutic, and clinical dimensions of heart failure therapy. By weaving molecular discoveries into therapeutic evaluation and clinical application, it advances both scientific understanding and practical strategies for improving patient outcomes.

This collection illustrates the breadth of current inquiry into heart failure, spanning molecular biology, therapeutic evaluation, and clinical practice. Mechanistic investigations have advanced understanding of disease progression. Liu et al. demonstrated that early treatment with nootkatone prevents pressure overload–induced ventricular remodeling and heart failure. Du et al. showed that Poge heart-saving decoction mitigates apoptosis and fibrosis via PI3 K/AKT regulation. Yongjie et al. introduced the concept of HIF-mediated hierarchical hypoxic adaptation as a novel paradigm in heart failure pathogenesis. Complementary studies addressed oxidative stress Zhang et al., inflammasome activation in HFpEF Yu et al., and genetic determinants of disease progression. These contributions broaden the mechanistic landscape, identifying molecular targets with therapeutic potential. Therapeutic evaluations highlight both established and innovative strategies. Interventions such as cardiac resynchronization therapy Małek-Elikowska et al. and beta-blockers Xie et al. were reassessed, while comparative analyses of angiotensin receptor–neprilysin inhibition provided evidence for optimizing pharmacological regimens. Emerging agents including vericiguat Yongjie et al. and finerenone Zhao et al. and Peng et al. demonstrated promise in improving outcomes. Reviews emphasized advances in mineralocorticoid receptor antagonists in the era of SGLT2 inhibitors and GLP-1 receptor agonists Wang et al., while novel approaches explored bioactive peptide-peptoid hybrids Kumar et al. and long-acting relaxin analogues. Opinion contributions advocated for integrating pharmacological and device-based strategies Li et al., underscoring the importance of multimodal approaches tailored to patient needs. Clinical perspectives highlight ongoing challenges in care delivery. Investigations addressed drug resistance Zhao et al., adverse effects Wang et al., and disparities in treatment accessibility. Several contributions emphasized individualized therapy Kumar et al., while perspectives on precision medicine and interdisciplinary collaboration reinforced the need to align basic science, clinical practice, and health policy. Additional studies explored multi-omics approaches for syndrome differentiation in ischemic heart failure Zhang et al. and identified biomarkers such as CLU, FOS, and CXCL8 for disease progression. Altogether, the 18 contributions provide a multidimensional view of heart failure therapy. By weaving mechanistic discoveries into therapeutic evaluation and clinical application, this collection advances both scientific understanding and practical strategies for improving patient outcomes.

The contributions to this collection underscore both the progress achieved and the challenges that continue to shape patient care. Beyond identifying pathways such as myocardial remodeling Liu et al., metabolic regulation Du et al., and mitochondrial–ion channel interactions Yongjie et al., these studies highlight how mechanistic discoveries can inform therapeutic innovation. Evaluations of interventions ranging from established therapies such as cardiac resynchronization Małek-Elikowska et al. and beta-blockers Xie et al. to newer agents including vericiguat Yongjie et al. and finerenone Zhao et al. and Peng et al. reveal encouraging outcomes, while also drawing attention to variability in patient response. Reviews emphasized advances in mineralocorticoid receptor antagonists in the era of SGLT2 inhibitors and GLP-1 receptor agonists. Persistent concerns, including drug resistance Zhao et al., adverse effects Wang et al., and inequities in access Yang et al., emphasize that therapeutic progress must be matched by practical solutions in care delivery. Looking ahead, the translational impact of these findings will hinge on bridging laboratory insights with clinical application. Precision medicine approaches that integrate genetic, metabolic, and phenotypic data Kumar et al. are poised to redefine individualized care. Successful adoption of novel agents and device-based strategies will depend on comparative effectiveness studies Cheng et al., long-term safety assessments, and frameworks that ensure equitable access. Future directions extend beyond therapy alone. Deeper exploration of molecular heterogeneity Zhang et al., integration of multi-omics data for patient stratification, and expansion of interdisciplinary collaborations that connect science, practice, and policy Li et al. will be essential. Pursuing these avenues can move the field closer to therapies that are not only effective but also sustainable and broadly accessible. In conclusion, this collection demonstrates that progress in heart failure therapy depends on the synergy of mechanistic insight, therapeutic innovation, and clinical pragmatism. The next frontier lies in ensuring that discoveries at the bench translate into meaningful improvements at the bedside, reshaping outcomes for patients worldwide.

